# Pharmaceutical Efficacy of Gypenoside LXXV on Non-Alcoholic Steatohepatitis (NASH)

**DOI:** 10.3390/biom10101426

**Published:** 2020-10-08

**Authors:** Jin Ha Lee, Ji Young Oh, Soo Hyun Kim, In Jeong Oh, Yong-ho Lee, Keun Woo Lee, Woong Hee Lee, Jeong-Hwan Kim

**Affiliations:** 1 MODNBIO Inc., digital road 34, Kolon Science Valley I, Guro-gu, Seoul 08378, Korea; jinha1118@modnbio.com (J.H.L.); leekw@modnbio.com (K.W.L.); xpress24@nate.com (W.H.L.); 2 Department of Internal Medicine, Yonsei University College of Medicine, 50–1 Yonsei-ro, Seodaemun-gu, Seoul 03722, Korea; iamojy@gmail.com (J.Y.O.); shkim6784@naver.com (S.H.K.); olib3068@gmail.com (I.J.O.); YHOLEE@yuhs.ac (Y.-h.L.); 3 Institute of Biotechnology, Chungnam National University, Daejeon 34134, Korea; 4 Cardiovascular Research Institute, Graduate School of Medicine, Yokohama City University, 3-9 Fukuura, Kanazawa-ku, Yokohama 236-0004, Japan

**Keywords:** ginsenosides, gypenoside LXXV, drug discovery, liver fibrosis, nonalcoholic steatohepatitis (NASH), hepatic stellate cells

## Abstract

Ginsenosides have offered a wide array of beneficial roles in the pharmacological regulation of hepatic metabolic syndromes, including non-alcoholic steatohepatitis (NASH), non-alcoholic fatty liver disease (NAFLD), and obesity. Of the numerous ginsenosides, Rg3 has been widely investigated, but there have been few studies of gypenosides (Gyp). Particularly, no study on Gyp LXXV has been reported to date. Here, to firstly explore the pharmacological effects of Gyp LXXV against NASH and the related mechanism, methionine- and choline-deficient (MCD) diet-induced NASH mice and hepatic cells (stellate cells, hepatic macrophages, and hepatocytes) were selected. Gyp LXXV exhibited markedly alleviated MCD diet-induced hepatic injury, inflammation, and fibrosis by down-regulating hepatic fibrosis markers such as α-smooth muscle actin(α-SMA), collagen1, transforming growth factors-β (TGF-β1), tumor necrosis factor-α (TNF-α), MCP-1, interleukin (IL)-1β, nuclear factor κB (NFκB), and GRP78. Remarkably, histopathological studies confirmed that 15 mg/kg of Gyp LXXV administration to MCD diet-induced mice led to effective prevention of liver injury, lipid accumulation, and activation of hepatic macrophages, indicating that Gyp LXXV might be a potential anti-NASH drug.

## 1. Introduction

Non-alcoholic steatohepatitis (NASH) is a severe liver disease that begins with fat accumulation in the organ, resulting in chronic liver injury, necrosis, inflammation, and unbalanced lipid metabolism in the liver, which can lead to hepatic fibrosis, dysfunction, and even cirrhosis which ultimately leads to cancer [1,2,3,4,5,6,7,8,9]. Particularly, obesity and insulin resistance are considered main factors in the initiation and perpetuation of NASH [10] and play a key role in the pathogenesis of type 2 diabetes mellitus and non-alcoholic fatty liver disease (T2DM-NAFLD) [11].

At the cellular level, liver damage through fat accumulation induces mitochondrial dysfunction, which can cause harmful effects on hepatic inflammation, reactive oxygen species (ROS) homeostasis, and cell death, resulting in NASH [12]. Simultaneously, it activates hepatic stellate cells (HSC) via progressive release of pro-inflammatory cytokines (platelet derived growth factor (PDGF), transforming growth factors-β (TGF-β), interleukin (IL)-1, tumor necrosis factor-α (TNF-α), and some chemokines) [13,14,15]. The activated HSCs multiply fibrogenic cells, generating a micro-environment favorable to cellular proliferation and survival by upregulating mesenchymal markers (α-smooth muscle actin (α-SMA, ACTA2), desmin (DES), and collagen α1(I)) [13,14,15].

TGF-β has been considered a major pro-fibrogenic cytokine and a promising target for treating hepatic fibrosis [15,16,17,18]. Although there have been many clinical reports focused on the importance of TGF-β activation in the pathogenesis of liver fibrosis in patients, its inhibition causes undesirable side effects, thereby decreasing the therapeutic advantages [15,17,18]. Therefore, understanding the pleiotropic effects of TGF-β and its regulatory mechanisms will help design better TGF-β-based therapeutics. Although there is no approved cure for NASH and a game-changing clinical approach has not yet been identified, several studies to ameliorate NASH are being performed in clinical trials [17,18]. In fact, an emerging trend in NAFLD or NASH treatment is represented by healthy lifestyle modifications, including diet and exercise [19].

As an active ingredient derived from natural products, ginseng ginsenoside has excellent therapeutic effectiveness with high bioavailability and little biotoxicity, and has been widely employed to treat various metabolic syndromes such as diabetes, liver diseases, heart diseases, cancer, etc [20,21,22,23,24]. A mixture of ginsenoside Rg3, Rg1, Rb1, and probiotics has been used as a putative treatment for NAFLD symptoms to diminish liver inflammation by decreasing the expression of IL-1β and phospho-p38 [23]. Ginsenoside Rg1 exhibited an anti-inflammatory effect on palmitic acid-induced HepG2 cells via the AMPK/NFκB pathway [24].

Gypenosides (Gyp) are a dammarane-type triterpene glycoside extracted from *Gynostemma (G.) pentaphyllum (Thunb.)* Makino possesses numerous pharmacological properties on metabolic diseases, including anti-aging, anti-oxidation, anti-inflammation, cardioprotective and neuroprotective effects, as well as wound healing properties [25,26,27]. Gyp extracts also have been shown to have a therapeutic effect of anti-hepatic lipogenesis [28,29]. Notably, a recent study showed that it alleviated NAFLD in mice by enhancing the intestinal probiotic property [29]. Furthermore, Gyp extracts exhibited a hepato-protective effect on T2DM-NAFLD by down-regulating the mRNA levels of tumor necrosis factor-α (TNF-α), nuclear factor κB (NFκB), peroxisome proliferator activated receptor (PPARγ), and cytochrome P4501A1 (CYP1A1) [30]. However, there is a limited number of reports and a lack of progress on drug screening and development of Gyp compounds since the regulatory mechanism on liver fat metabolism of Gyp compounds’ action in targeting NASH is still uncertain. In this study, for the first time, we deliver compelling evidence on the therapeutic efficacy of a Gyp LXXV (one of the Gyp compound types) compound against high-fat diet-induced NASH, finding the potential mechanisms involved in liver fibrogenesis.

## 2. Materials and Methods

### 2.1. Materials

Fetal bovine serum (FBS), Dulbecco’s modified Eagle’s medium (DMEM), Roswell Park Memorial Institute (RPMI)-1640 culture medium and penicillin–streptomycin were purchased from GE Healthcare Life Sciences (Marlborough, MA, USA). IL-1β ELISA kit (Human 88-7261-22) and TRIzol reagent (15596018) was purchased from Invitrogen (Carlsbad, CA, USA). Lipopolysaccharide (LPS; L6529), palmitate (P9767) and MCC950 (PZ0280) were obtained from Sigma-Aldrich (St. Louis, MO, USA). Pioglitazone was purchased from Takeda Pharmaceutical (AD-4833, Kanagawa, Japan). Ezetimibe was obtained from Cayman Chemical (16331, Ann Arbor, MI, USA). The triglyceride assay kit was purchased from Bioassay systems (ETGA-200, Hayward, CA, USA). Anti-F4/80 antibody was obtained from Abcam (ab6640, Cambridge, MA, USA). 3-(4,5-dimethylthiazol-2-yl)-2,5-diphenyltetrazolium bromide (MTT) was purchased from Georgiachem (MT 1036, Norcross, GA, USA).

### 2.2. Characterization of High-Purity Gyp LXXV and Ginsenoside Rg3 Compounds

The Gyp LXXV and ginsenoside Rg3 compounds were prepared at MODNBIO Inc. (Seoul, Korea) and analyzed by HPLC systems that were controlled by LabSolutions^®^ software (Shimadzu, Japan) running under Microsoft Windows 7. Each compound was dissolved in a methanol solution, and 20 μL of the solution was injected into the column (ODS, 250 × 4.6 mm, 150 Å). The flow rate was 1.0 mL/min, and the UV–vis absorbance peaks were measured at 203 nm.

### 2.3. Cell Culture and Viability Assay

Hepatic cell line HepG2 and hepatic stellate cell line LX2 were cultured with DMEM supplemented with 10% FBS and 1% penicillin–streptomycin solution, and they were grown in 100 mm culture dishes in a 5% CO_2_ incubator at 37 °C. THP-1 cells were cultured with RPMI-1640 culture medium supplemented with 10% FBS and 1% penicillin–streptomycin solution and were grown in 75 cm^2^ flasks. The cytotoxicity in cultured cells was investigated through MTT assay (tetrazolium-based colorimetric). Cells were treated with either Gyp LXXV or Rg3. After treatment, the cells were incubated with 5 mg/mL MTT reagent for 1 h at 37 °C in the dark. MTT reagent was aspirated, and dimethyl sulfoxide was added to each well to dissolve MTT formazan crystals. Absorbance was measured at 560 nm using a multiple plate reader.

### 2.4. ELISA for IL-1β-Detection

After stimulating the THP-1 cells with macrophages, the cultured supernatant was collected, and then IL-1β concentration was determined using an IL-1β ELISA kit (Thermo Fisher Scientific, 88-7261-88) according to the manufacturer’s instructions.

### 2.5. RNA Isolation and Reverse Transcription–Polymerase Chain Reaction (RT-PCR)

Total RNA was isolated using TRIzol reagent, and then cDNA was synthesized from the total RNA using the High-Capacity cDNA Reverse Transcription kit (Applied Biosystems, 4368814, Waltham, MA, USA). The cDNA was mixed with the Power SYBR^®^ Green PCR Master Mix (Applied Biosystems, 4367659, Waltham, MA, USA) and primers, and amplified in StepOne^TM^ Real-Time PCR Systems (Applied Biosystems, Waltham, MA, USA).

### 2.6. Animal Preparation

Seven-week-old male C57BL/6J mice weighing 20–25 g were purchased from Japan SLC, Inc. (Hamamatsu, Shizuoka, Japan). Mice were acclimatized seven days before the start of the experiment in the facility under standard environmental conditions (23 ± 2 °C, 12/12 h light/dark cycle with lights on at 08:00) and had ad libitum access to food and water. All animal care and procedures were performed in accordance with Yonsei University College of Medicine guidelines and approved by the institutional animal care and use committee at Yonsei University College of Medicine (#2018-0275).

### 2.7. Animal NASH Model

Mice were fed a normal diet (vehicle) or a methionine- and choline-deficient (MCD) L-amino acid diet (Research Diet, Inc., A02082002BR, NJ, USA) for 6 weeks. The mice were randomly divided into seven groups as vehicle, MCD, Rg3 (15, 30 mg/kg/daily), Gyp LXXV (15, 30 mg/kg/daily) and MCC950 (positive control). Each sample was orally administrated during the last 3 weeks of the 6 weeks of being fed the MCD diet. After administration of the compounds for 3 weeks, the MCD mice were euthanized through cardiac blood collection; following this, the liver tissue was sampled.

### 2.8. Blood Chemistry Assay

Serum was isolated from the blood collected from the mice using centrifugation at 3000 rpm for 15 min. Alanine aminotransferase (ALT) (Fujifilm, 3250) and aspartate aminotransferase (AST) (Fujifilm, 3150) in serum were analyzed using Fuji dry-chem 4000i (Fujifilm, Barcelona, Spain).

### 2.9. Immunohistochemistry (IHC)

Paraffin-embedded tissue sections were deparaffinized with xylene, and then rehydrated in serial dilutions of ethanol. The tissues were washed using distilled water and were then heated in antigen retrieval by sodium citrate buffer (pH 6). The tissues were incubated with hydrogen peroxidase blocking solution (S2023, Dako, Denmark) for 10 min at room temperature (RT) and were treated with protein blocking solution for 20 min at RT. The tissues were incubated at 4 °C overnight with a monoclonal anti-F4/80 antibody (1:100 dilution) or a polyclonal anti-α-SMA antibody (1:100 dilution). The slides were incubated for 30 min with a secondary antibody and then incubated with avidin–biotin complex reagent for 30 min at RT. The tissues were developed with 3,3’Diaminobenzidine (DAB) substrate and counterstained with hematoxylin. IHC staining intensities were analyzed using Fiji software (National Institutes of Health, Maryland, MA, USA). F4/80- or α-SMA-positive cells were normalized by nuclei.

### 2.10. Oil Red O Staining

For frozen sections, liver tissues were fixed with 4% paraformaldehyde overnight and then infiltrated in 30% sucrose. The tissues were embedded in OCT compound and stored at −80 °C until analysis. Frozen tissues were sectioned at 6 μm using a cryostat (CM1860, Leica Biosystems, IL, USA). The sections were air-dried for 30 min and fixed in 10% formalin for 5 min. The slides were washed with 60% isopropanol and then stained with oil red O (Sigma-Aldrich, O0625) working solution for 15 min. The slides were counterstained with hematoxylin and washed with distilled water.

### 2.11. Western Blot Analysis

Cells and liver tissues were lysed with RIPA buffer (ATTO Corporation, Tokyo, Japan) and quantified using a BCA protein assay kit (Thermo Fisher Scientific, Waltham, MA, USA). Proteins were separated by SDS-PAGE and transferred to a polyvinylidene difluoride membrane. The membrane was incubated with 5% skim milk for blocking, and then washed with Tris-buffered saline, 0.1% Tween 20 (TBST). The membrane was incubated with primary antibodies against α-SMA (Abcam, ab5694), p-NFκB (Cell Signaling, 3033S), NFκB (Santa Cruz, sc-372), Glyceraldehyde 3-phosphate dehydrogenase (GAPDH) (Santa Cruz, sc-25778) overnight at 4 °C. Specific bands were visualized by a Chemi-luminescent Image Analyzer (ImageQuant^TM^ LAS 4000, Fujifilm, Tokyo, Japan) and analyzed by Image J software.

### 2.12. Hepatic Triglyceride (TG) Assay

After 30 mg of liver tissue was homogenized, the analysis of hepatic TG was performed using the EnzyChrom^TM^ Triglyceride Assay Kit (ETGA-200, BioAssay Systems, Waltham, CA, USA) according to the manufacturer’s instructions.

### 2.13. Statistics

Data were presented as mean ± standard error of mean (SEM). The results were analyzed by unpaired two-tailed Student’s t-test.

## 3. Results and Discussion

### 3.1. Characterization of High-Purity Gyp LXXV and Ginsenoside Rg3 Compounds

The HPLC chromatogram clearly confirmed the high-quality (~99%) single signatures of Gyp LXXV and Rg3 samples (Figure S1). The solubility of the compounds was confirmed using a turbidity method prior to all the in vitro and in vivo studies, as reported in the previous study [31].

### 3.2. Gyp LXXV Inhibits the TGF-β-Induced Activation of Hepatic Stellate Cells

**Figure 1:**
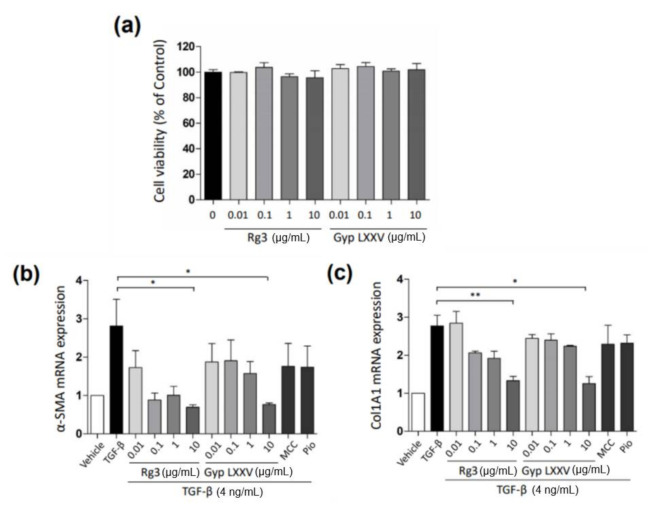
Effect of gypenoside LXXV (Gyp LXXV) on cell viability and fibrosis in hepatic stellate cells (LX-2). Cell viability of Gyp LXXV or Rg3 on LX-2 cells (**a**). Cells were treated with increasing concentrations of Gyp LXXV or Rg3 (0.01–10 μg/mL) for 24 h. Gyp LXXV-mediated inhibition of fibrosis markers, α-smooth muscle actin (α-SMA) (**b**) and collagen1 (**c**), via transforming growth factors-β (TGF-β) (4 ng/mL)-induced LX-2 activation (n = 3 for each group). * *p* < 0.05, ** *p* < 0.01 vs. TGF-β. MCC950 (MCC; 1 μM) or pioglitazone (Pio; 10 μM) was used as positive controls.

Throughout chronic liver injury, TGF-β plays a prominent role in stimulating liver fibrogenesis by myofibroblast-like cells derived from HSCs. To assess the potential inhibitory effect of Gyp LXXV against TGF-β-induced HSCs, the in vitro inhibitory rate of the compounds was monitored in LX-2, a well-established HSC cell line. The cell viability of Gyp LXXV in the LX-2 cells was confirmed by MTT assays, showing no cytotoxicity at all concentrations (Figure 1a). To evaluate the regulatory effects of Gyp LXXV in the TGF-β-induced activation of LX-2, the expressions of fibrosis markers (α-SMA and col1agen1) were examined by a RT-PCR analysis. In Figure 1b,c, following treatment with TGF-β1 to induce a fibrosis, the RNA expressions of α-SMA and col1agen1 in LX-2 were dramatically increased compared to the control. The Gyp LXXV treatment significantly reduced the production of α-SMA and col1agen1 in a dose-dependent manner. The results were similar to those obtained when cells were exposed to Rg3 used as a ginsenoside control treatment.

Furthermore, MCC950, a selective inhibitor of NLRP3 inflammasome, has been used to treat the liver inflammation and fibrosis [32]. In this work, we employed MCC950 as a positive control in in vivo experiments to confirm the effects of Gyp LXXV or Rg3 on improving efficacy of hepatic fibrosis and inflammation in the MCD-fed mouse model. In addition, pioglitazone is currently being used in NASH patients with type 2 diabetes, which improves liver injury and fibrosis. Ezetimibe, a lipid-lowering agent, has been reported to improve hepatic steatosis, inflammation, and fibrosis [33]. Accordingly, pioglitazone, ezetimibe, or MCC95 were used as positive controls in this work.

### 3.3. Gyp LXXV Inhibits the TGF-β-Induced Activation of Hepatocytes (HepG2 Cells)

We performed the cell viability assay of the compounds using hepatocytes, HepG2 cells. As presented in Supplementary Information (Figure S2), an alleviated effect against a palmitate-induced cell death was significantly enhanced by either administration of Rg3 or Gyp LXXV in the cells.

### 3.4. Inflammasome Activity in Hepatic Macrophages (THP-1 Cells)

**Figure 2:**
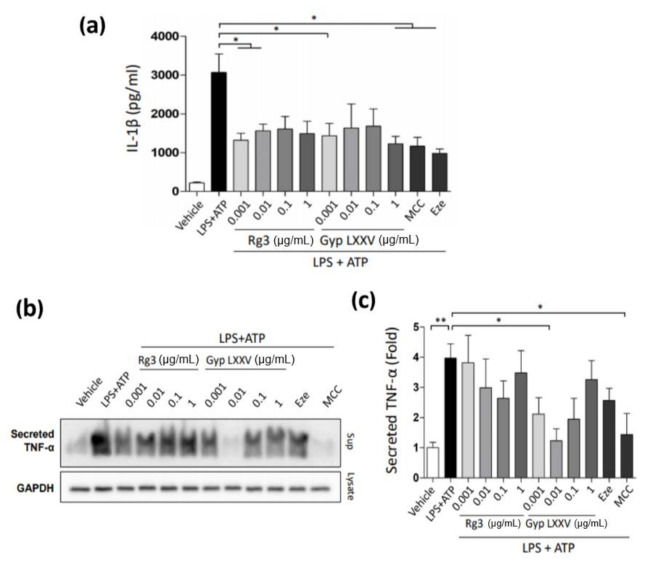
Inhibition effect of Gyp LXXV on inflammation using macrophage-induced THP-1 cells. Attenuated secretion level of IL-1β (**a**) or TNF-α (**b**, **c**) for Gyp LXXV or Rg3 at various concentration (0.001–1 μg/mL). THP-1 cells were stimulated with lipopolysaccharide (LPS) (0.1 μg/mL) and ATP (2 mM). * *p* < 0.05 vs. LPS + ATP group. MCC (10 nM) or ezetimibe (Eze; 50 μM) was used as a positive control.

Hepatic macrophages play an important role in keeping hepatic immune homeostasis [34,35,36]. Their chief function in the pathogenesis of NASH has made them an attractive therapeutic target for NASH treatment [35,36]. To examine the potential effect of macrophage-mediated inflammation in NASH, the hepatic macrophages, THP-1, were treated with LPS + ATP to induce an inflammasome activity. Then, the Gyp LXXV or Rg3 compounds were treated on the THP-1 cells. While the stimulation of THP-1 with LPS and ATP increased secretion of IL-1β (Figure 2a) or TNF-α (Figure 2b,c), the treatment of Rg3 or Gyp LXXV substantially attenuated the secretion levels, indicating that the compounds could inhibit the activation of inflammasome. Particularly, 10 ng/mL of Gyp LXXV substantially attenuated the activation level of LPS + ATP-stimulated TNF-α that was increased at the higher concentration of the compound.

There are many reports that NOD-like receptor protein 3 (NLRP3) inflammasome activation in NAFLD causes liver inflammation [37,38,39]. However, to better understand macrophage cells to develop novel macrophage-based therapeutic interventions in hepatic inflammation, more research on their phenotypes and functions is required. To further confirm the pro-inflammatory profile in hepatic macrophages, we investigated the effects of Gyp LXXV or Rg3 on the mRNA levels of pro-inflammatory genes induced by LPS in THP-1 cells (Figure S3). The LPS-induced TNF-α expression was reduced by Gyp LXXV or Rg3 treatment, while IL-10 expression was unchanged.

### 3.5. Effects of Gyp LXXV on Inflammation and Endoplasmic Reticulum (ER) Stress in Hepatocytes (HepG2 Cells)

To examine an inhibitory effect of inflammation and ER stress in hepatocytes, HepG2 cells were induced with palmitate (0.01, 0.1, 1, 10 μg/mL) and treated with the Gyp LXXV or Rg3 compounds onto the cells. It was confirmed that both compounds exhibited noticeable inhibition effect against TNF-α from the palmitate-induced cells, while showing slight inhibitory effect against GRP78 from the cells (Figure S4).

### 3.6. Effect of Gyp LXXV on NASH in Methionine- and Choline-Deficient (MCD) Diet-Induced Mice

The MCD diet is a common reproducible model to gradually induce the phenotype of severe NASH, including steatosis, hepatic inflammation, and fibrosis after administration, which induces NAFLD/NASH, causing histologically close liver damage to human NASH [40].

**Figure 3:**
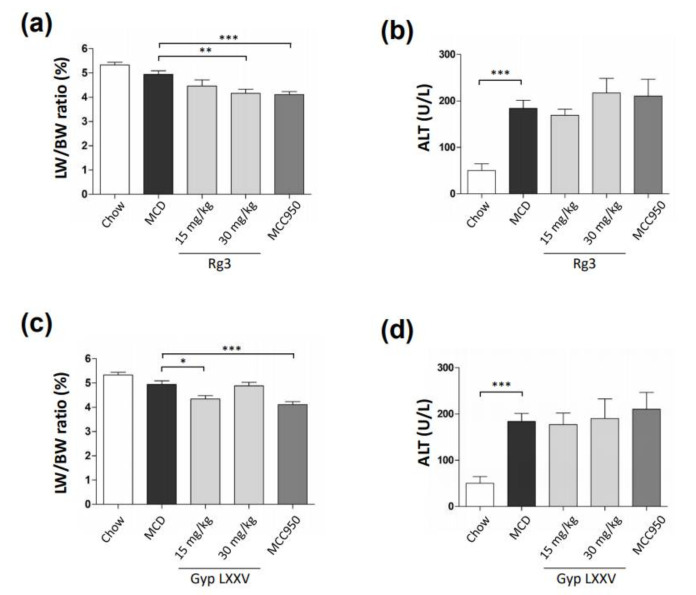
Effect of Rg3 (**a**,**b**) or Gyp LXXV (**c**,**d**) on the liver-to-body weight ratios (LW/BW ratio) and serum enzyme levels (ALT) in the MCD diet mouse model. Error bars represent SEM. * *p* < 0.05, ** *p* < 0.01, *** *p* < 0.001 vs. the MCD group, two tailed unpaired t-test.

To evaluate the effect of Gyp LXXV on non-alcoholic steatohepatitis (NASH), we fed mice with the methionine- and choline-deficient (MCD) diet for 6 weeks. After oral administration of the compounds, the body weight of mice was measured every week. There was no weight change in groups of Gyp LXXV as well as Rg3 compared to the MCD diet mouse group that showed a significant decrease in weight (Figure S5). It was confirmed that the liver tissue/weight ratio of the group administered with each compound was significantly reduced compared to the MCD group (Figure 3a,c). The serum levels of alanine aminotransferase (ALT) and aspartate aminotransferase (AST) are surrogate markers for liver injury. When Rg3 was administered in the MCD diet model, the serum levels of ALT and AST were confirmed to be comparatively unchanged compared to the MCD group (Figure 3b). When Gyp LXXV was fed, the levels were not changed compared to the MCD group, excluding the increased level (< ~800 U/L) in the group of 60 mg/kg dosed (Figure 3d), which is unharmed since a hepatotoxicity is defined as AST or ALT concentrations greater than 1000 IU/L.

**Figure 4:**
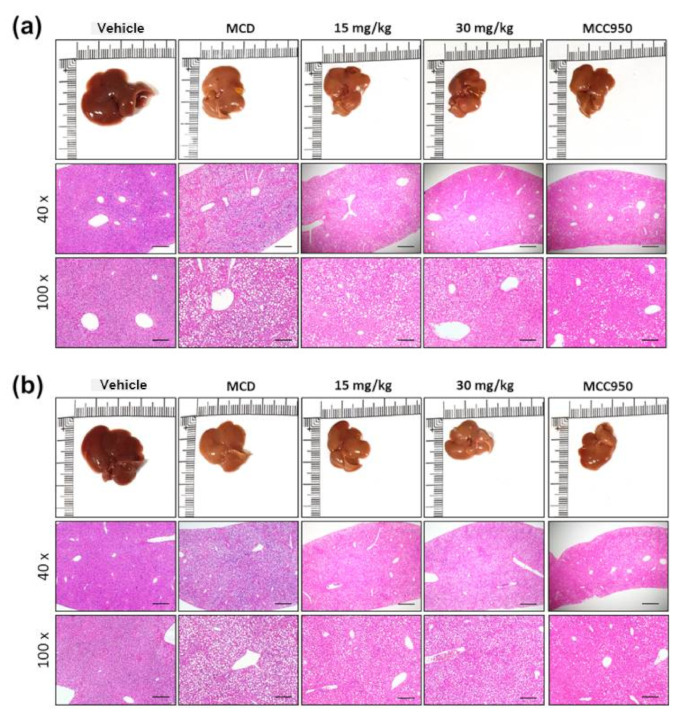
Alleviating effect of Rg3 or Gyp LXXV on fatty liver and hepatitis in the MCD diet mouse model. Representative photographs of the fresh livers and histopathological haematoxylin eosin (H&E) staining of liver sections administered Rg3 (**a**) or Gyp LXXV (**b**) (15–30 mg/kg) are presented the middle panels with a 40× magnification and a scale bar 500 μm, and the lower panels with a 100× magnification and a scale bar 200 μm.

A histological study also provided evidence supporting the results of the serum biochemical analysis. As shown in Figure 4, MCD diet-treated mice had obvious vesicular lesions and severe neutrophil infiltration. In animals treated with MCC950, greatly reduced fat droplets were exhibited when compared to the free MCD model. Consistently, Gyp LXXV and Rg3 displayed significant reductions of fat droplets compared to the free MCD model. Extraordinarily, at an equal level of a low dose range (15 mg/kg), the reduction rate of fat accumulation in the Gyp LXXV-treated group (Figure 4a) was obviously greater than that of the Rg3-treated group (Figure 4b), which allows prevention of MCD diet-induced liver injury and lipid accumulation. Moreover, this meets the preferred condition, meaning that it is safe to pursue low dosage-based administration to avoid any potential hepato-cytotoxicity and side effects when compared with results elsewhere on significantly high oral doses of Gyp extracts (200–800 mg/kg) to treat T2DM-NAFLD [30].

**Figure 5:**
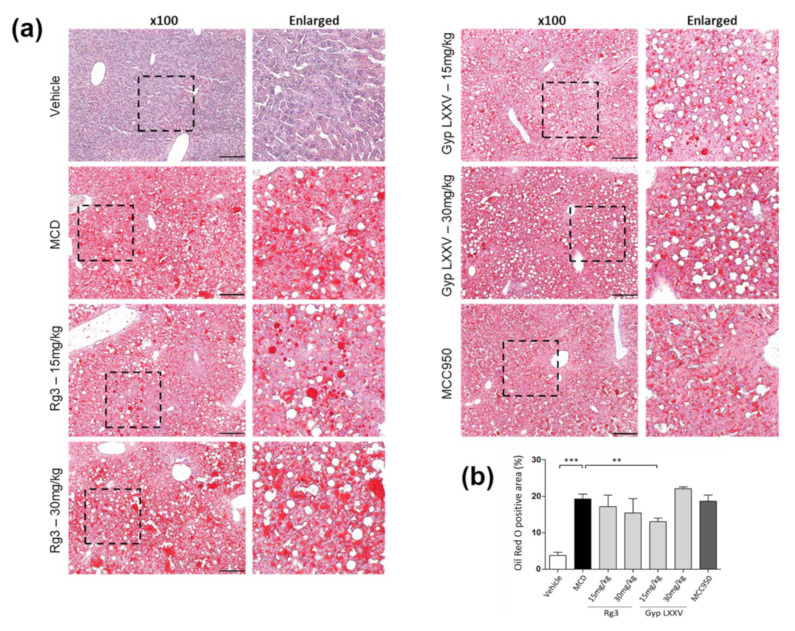
Oil red O staining of liver sections from the control and MCD-induced mice groups to validate the ameliorating effect of Gyp LXXV against hepatic lipid accumulation. (**a**) 100x magnification, scale bar 200 μm. Quantification of oil red O staining was performed using Fiji software (**b**). Error bars represent SEM. ** *p* < 0.01, *** *p* < 0.001 vs. MCD, two tailed unpaired t-test.

To confirm the lipid accumulation in the liver of MCD-fed mice, oil red O staining was used to measure fat loading in the hepatocytes (Figure 5a). Histological analysis revealed an increased intracellular lipid deposition in the liver of MCD group. The lipid droplets were markedly reduced in the livers of Rg3- or Gyp LXXV-treated mice. In particular, the reduction rate of fat accumulation in the Gyp LXXV-treated group at a low dose range (15 mg/kg) was apparently greater than that of the Rg3-treated group, as quantitated in Figure 5b. We have also monitored the level of lipid metabolic parameters such as triglycerides (TG), which remained unchanged (Figure S6). It is assumed that Gyp LXXV may be effective in other lipid types in NASH-associated lipid metabolism other than TG.

### 3.7. Effect of Gyp LXXV in Hepatic Fibrosis in MCD Diet-Induced Mice

Hepatic fibrosis is an advanced liver disease state that could develop into hepatocellular carcinoma, characterized by the net accumulation of extracellular matrix resulting from NASH [9,14,15]. However, there is no direct approved anti-fibrotic therapy, and existing treatment is mostly the invasive elimination of the associated factors, e.g., surgery or transplantation [14].

**Figure 6:**
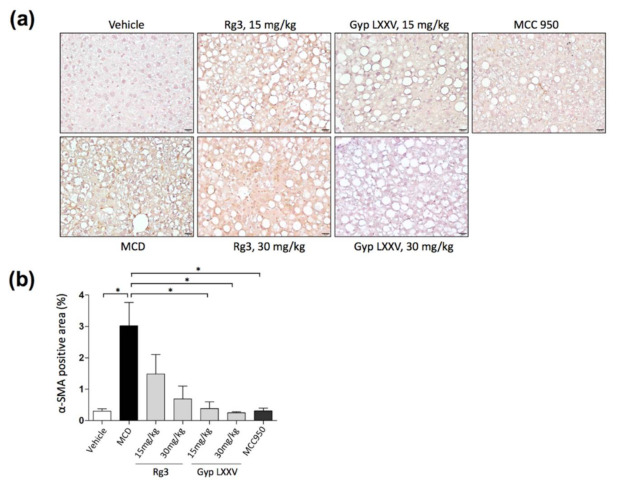
Immunohistochemical staining of α-SMA in liver samples (**a**). The distribution of hepatic fibrosis in the MCD diet mouse model with or without Gyp LXXV or Rg3 was investigated. 400× magnification, scale bar 20 μm. Quantification of immunohistochemistry (IHC) for α-SMA was performed using Fiji software (**b**). Error bars represent SEM. * *p* < 0.05 vs. MCD, two tailed unpaired t-test.

To confirm whether Gyp LXXV suppressed the activation of hepatic fibrosis in HSCs in vivo, we performed immunohistochemistry (IHC) for α-smooth muscle actin (α-SMA), a well-characterized fibrosis marker. As presented in IHC images (Figure 6a) and the quantitative data of α-SMA (Figure 6b), the distribution of hepatic fibrosis was substantially reduced in mice treated with Gyp LXXV compared to the group treated with Rg3, as well as mice fed MCD diet.

**Figure 7:**
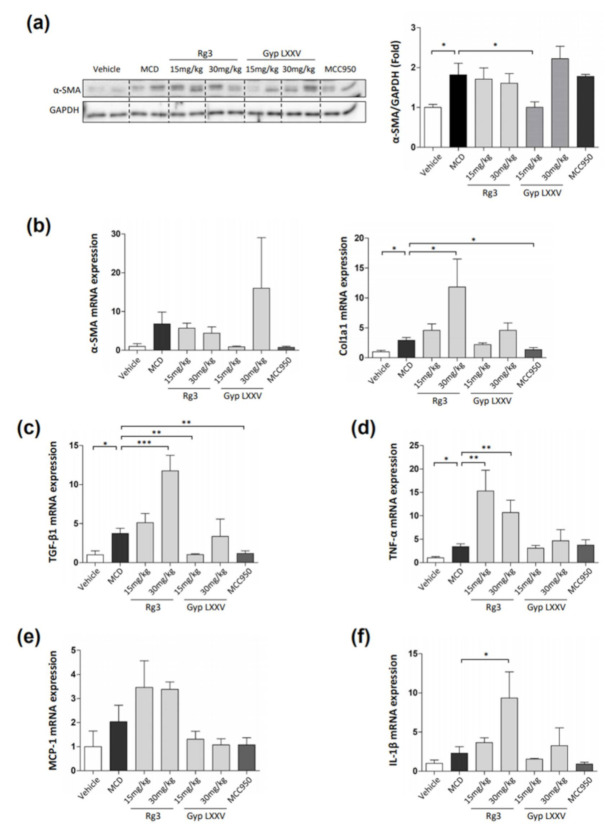
Inhibitory effect of Gyp LXXV on hepatic fibrosis in the MCD diet mouse model. The Western blot analysis of the liver tissues (left) to quantify α-SMA/GAPDH (right) (**a**). Table 1 a1, TGF-β1, TNF-α, MCP-1, and IL-1β in liver tissues were examined using a RT-qPCR (**b**–**f**). MCC950 (20 mg/kg) was used as a positive control. The error bars represent SEM. * *p* < 0.05, ** *p* < 0.01, *** *p* < 0.001 vs. MCD, two tailed unpaired t-test.

We also examined the hepatic mRNA expression of activation markers. As presented in Figure 7a, Western blot analysis revealed that the MCD group showed a more increased α-SMA expression than the vehicle group, while this level was significantly reduced by administration of Gyp LXXV (15 mg/kg). The mRNA expression of α-SMA and Col1a1 were reduced in mice treated with 15 mg/kg Gyp LXXV compared to mice fed MCD diet, but it was not statistically significant (Figure 7b). As shown in Figure 7c, the administration of 15 mg/kg Gyp LXXV remarkably reduced the mRNA expression of TGF-β1 to a level similar to the vehicle levels in the MCD-fed mouse model while exhibiting no change in the expression levels of TNF-α, MCP-1, and IL-1β (Figure 7d–f). Remarkably, treatment with 15 mg/kg of Gyp LXXV resulted in substantial declines in the expression level of all the markers, corresponding to the H&E staining results of the liver sections. Henceforth, these data imply that a low dose of Gyp LXXV can inhibit hepatic fibrosis in vivo. Notably, an increase in TGF-β1 expression is considered as a key driver in HSC activation to aggravate liver fibrosis [16,17]. As presented in Figure 7c, the expression of the TGF-β1 marker was prominently induced in the mouse model of liver fibrosis. The oral administration of Gyp LXXV significantly reduced the upregulation of hepatic TGF-β1 proteins.

### 3.8. Effect of Gyp LXXV on the Activation of Liver Macrophages in MCD Diet Models

**Figure 8:**
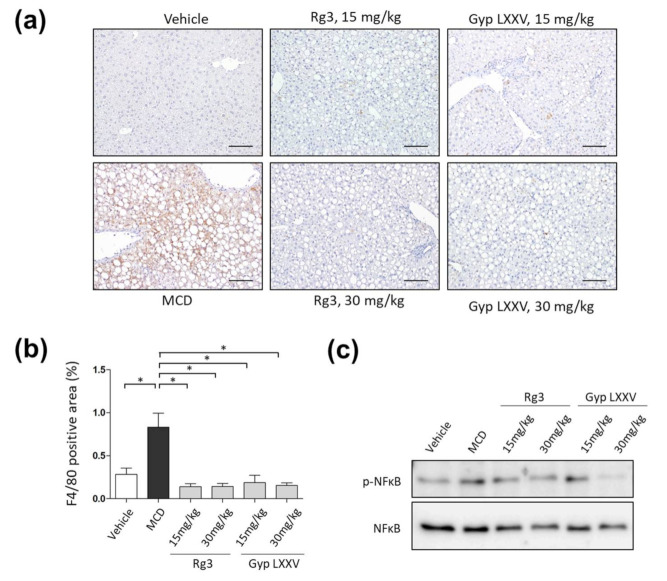
Inhibitory effect of Gyp LXXV on hepatic macrophages in the MCD diet mouse model. Representative IHC staining for F4/80 in the liver (**a**). The distribution of intracellular hepatic macrophages in the MCD diet mouse model with or without Gyp LXXV or Rg3 was investigated. 200× magnification, scale bar 100 μm. Quantification of F4/80 positive cells in paraffin sections of the liver (**b**). Error bar represent SEM. * *p* < 0.05 vs. MCD group, two tailed unpaired t-test. The protein levels of p-NFκB and nuclear factor κB (NFκB) were determined by Western blot analysis (**c**).

Macrophages are among the main inflammatory cells that play a role in inducing hepatitis [34]. Subsequent activation of Kupffer cells (KCs) and inflammasomes promotes massive release of pro-inflammatory, pro-fibrotic cytokines and ligands. The HSC is then stimulated to produce a large amount of extracellular matrix that causes progressive fibrosis [35]. The effect of Gyp LXXV on the activation of KCs in the liver was confirmed in the MCD mouse model through liver tissue F4/80 staining (Figure 8a). Compared to the MCD model, the quantitative effect of alleviating KC accumulation in the liver was observed in all the Gyp LXXV- and Rg3-treated groups (Figure 8b). Nuclear factor κB (NFκB) in macrophages could lead to liver inflammation and fibrosis, which regulates the inflammatory state and plays a key role in immune response [35]. Consistently with the IHC results, our Western blot analysis results have revealed that the level of p-NFκB expression of the group fed with either Rg3 or Gyp LXXV was significantly reduced compared to the MCD-fed group (Figure 8c), suggesting that the compounds may be an effective regulatory pathway into the activation towards liver fibrosis, enabling protection of NASH.

**Figure 9:**
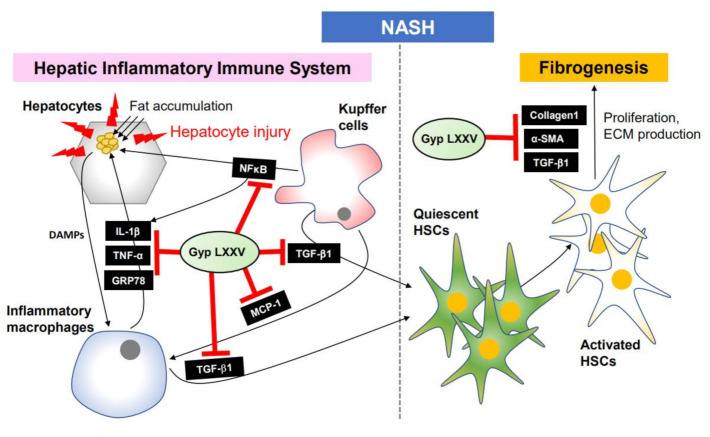
Schematic hepatic inflammation-dependent regulatory mechanism of Gyp LXXV against non-alcoholic steatohepatitis (NASH).

Taken together, the molecular inhibition mechanism of NASH signaling pathway by Gyp LXXV molecules is proposed in Figure 9. The hepatocyte injury by fat accumulation activates macrophages and HSCs through some cytokines (TGF-β1, IL-1β, and TNF-α), NFκB, and ER stress markers (GRP78), along with proliferation of fibrogenic cells by the activated HSCs in the presence of α-SMA, collagen1, TGF-β1, TNF-α, MCP-1, and IL-1β. Gyp LXXV compounds alleviate hepatic injury, inflammation, and fibrosis by down-regulating the hepatic fibrosis pathway, enabling a potential anti-NASH drug.

## 4. Conclusions

NASH is characterized by hepatocellular injury accompanied by steatosis, inflammation, and fibrosis, which should be targeted by modulating the signaling pathway to prevent and treat liver fibrosis as early as possible prior to further progression. In this study, Gyp LXXV, a natural ginseng extract-based potent small-molecule inhibitor, efficiently reduces liver lipid accumulation in MCD diet-induced mice. Moreover, it inhibited the progression of liver fibrosis by suppressing the activation of the fibrogenesis signaling pathway by blocking regulatory proteins to lessen liver fibrosis, implying that it is a novel and potential therapeutic strategy for antifibrotic intervention of NASH. Although the efficacy of the compounds was superior to the control groups, it was not concentration dependent. To move onto the clinical trials, further studies such as pharmacokinetics, pharmacodynamics, and safety remain to be explored.

## Supplementary Materials

The following are available online at https://www.mdpi.com/2218-273X/10/10/1426/s1, Figures S1–S6.
