# Supplementary material for: Pharmaceutical Efficacy of Gypenoside LXXV on Non-Alcoholic Steatohepatitis (NASH)
Source: Biomolecules. 2020 Oct 8;10(10):1426. doi: 10.3390/biom10101426 (PMC7599508; doi:10.3390/biom10101426)
Supplement: Supplementary file 1 [file biomolecules-10-01426-s001.pdf]

# Supplementary Information

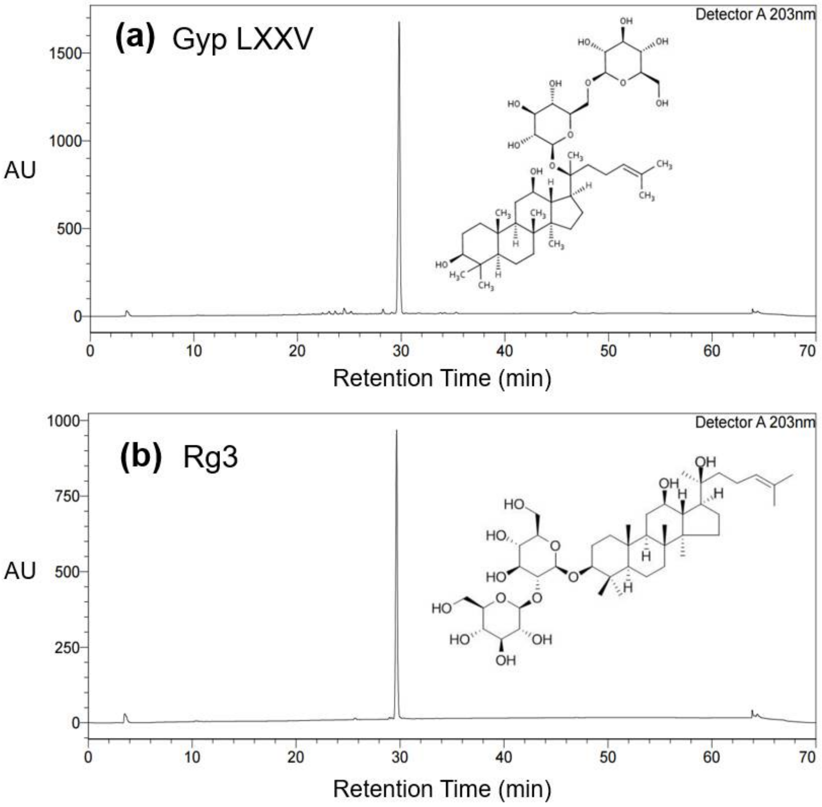


**Figure S1.** HPLC chromatograms of high-quality Gyp LXXV and Rg3 samples.

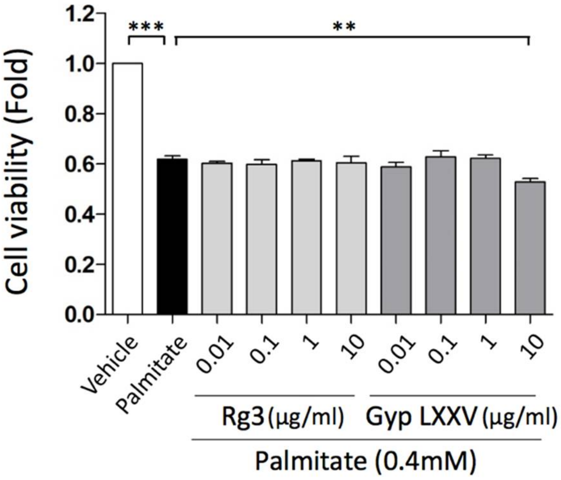


**Figure S2.** Effect of Gyp LXXV or Rg3 on cell viability in HepG2 cells. Cells were treated with either palmitate (0.4 mM) or palmitate in various concentration of Rg3 or Gyp LXXV (0.01–10 μg/mL) for 24 h, followed by a MTT assay. N = 4. Error bars represent SEM. $^{**}p < 0.01$, $^{***}p < 0.001$ vs. palmitate, two tailed unpaired t-test.

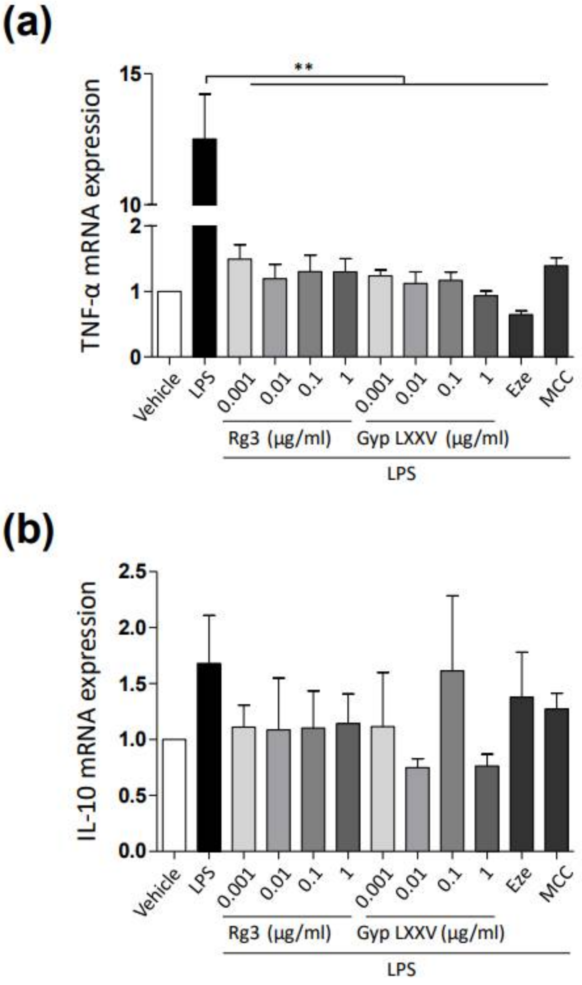


**Figure S3.** Effect of Gyp LXXV or Rg3 in the pro-inflammatory profile in THP-1 cells. Cells were treated with either LPS (0.1 μg/mL) or LPS in various concentration of Rg3 or Gyp LXXV (0.001–1 μg/mL). The mRNA expression of TNF-$\alpha$ and IL-10 were examined using RT-qPCR. Eze (50 μM) and MCC950 (10 nM) were used as positive controls. Error bars represent SEM. **$p < 0.01$ vs. LPS, two tailed unpaired t-test.

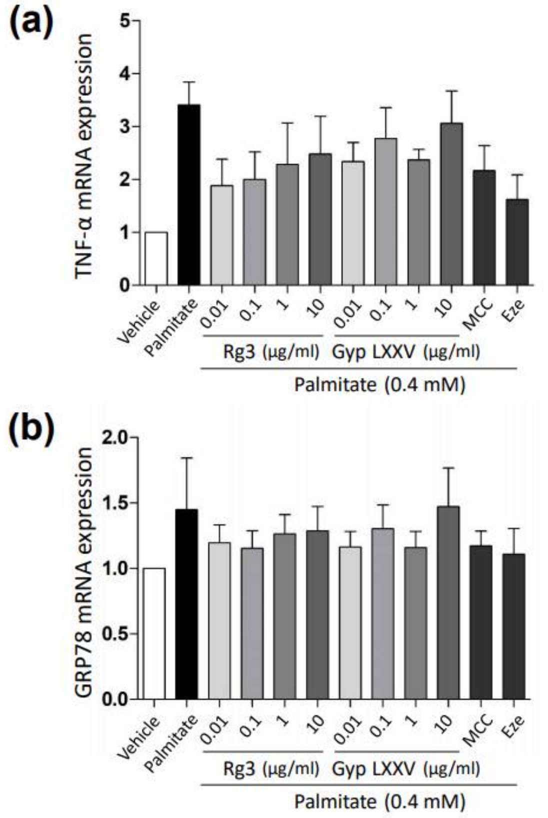


**Figure S4.** Effect of Gyp LXXV or Rg3 in inflammation and ER stress in HepG2 cells. Cells were treated either palmitate (0.4 mM) or palmitate with various concentration of Rg3 or Gyp LXXV (0.01–10 μg/mL). The mRNA expression of TNF-$\alpha$ and GRP78 were examined using a RT-qPCR. MCC950 (10 μM) and Eze (50 μM) were used as positive controls.

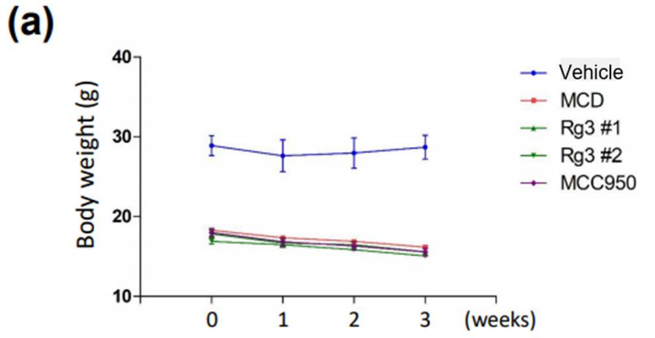


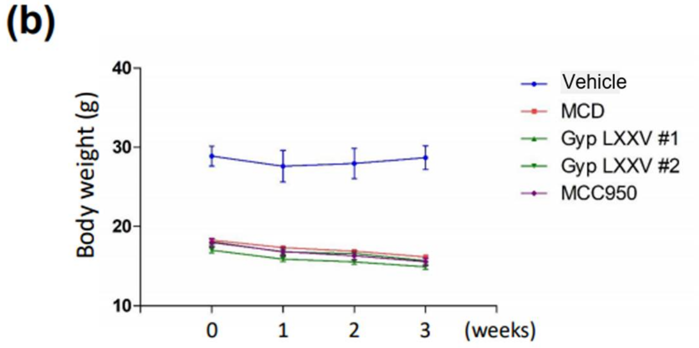


**Figure S5.** Body weight measurement of MCD diet mice after oral administration of Rg3 (**a**) or Gyp LXXV (**b**). Rg3 #1 (15 mg/kg), Rg3 #2 (30 mg/kg), Gyp LXXV #1 (15 mg/kg), Gyp LXXV #2 (30 mg/kg) and MCC950 (positive control, 20 mg/kg).

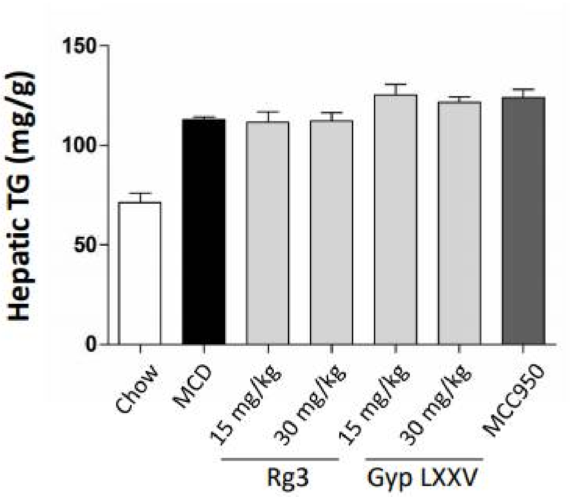


**Figure S6.** The effect of Gyp LXXV or Rg3 on hepatic TG in the MCD diet mice model.
